# Number Conservation is Related to Children’s Prefrontal Inhibitory Control: An fMRI Study of a Piagetian Task

**DOI:** 10.1371/journal.pone.0040802

**Published:** 2012-07-16

**Authors:** Nicolas Poirel, Grégoire Borst, Grégory Simon, Sandrine Rossi, Mathieu Cassotti, Arlette Pineau, Olivier Houdé

**Affiliations:** 1 Laboratoire de Pychologie du Développement et de l’Éducation de l’enfant (LaPsyDÉ, Unité CNRS 3521), Université Paris Descartes, Sorbonne Paris Cité, Paris, France; 2 Laboratoire de Pychologie du Développement et de l’Éducation de l’enfant (LaPsyDÉ, Unité CNRS 3521), Université de Caen, Caen, France; 3 Institut Universitaire de France (IUF), Paris, France; 4 Centre de Gestion Scientifique, Mines ParisTech, Paris, France; George Mason University/Krasnow Institute for Advanced Study, United States of America

## Abstract

Although young children can accurately determine that two rows contain the same number of coins when they are placed in a one-to-one correspondence, children younger than 7 years of age erroneously think that the longer row contains more coins when the coins in one of the rows are spread apart. To demonstrate that prefrontal inhibitory control is necessary to succeed at this task (Piaget’s conservation-of-number task), we studied the relationship between the percentage of BOLD signal changes in the brain areas activated in this developmental task and behavioral performance on a Stroop task and a Backward Digit Span task. The level of activation in the right insula/inferior frontal gyrus was selectively related to inhibitory control efficiency (i.e., the Stroop task), whereas the activation in the left intraparietal sulcus (IPS) was selectively related to the ability to manipulate numerical information in working memory (i.e., the Backward Digit Span task). Taken together, the results indicate that to acquire number conservation, children’s brains must not only activate the reversibility of cognitive operations (supported by the IPS) but also inhibit a misleading length-equal-number strategy (supported by the right insula/inferior frontal gyrus).

## Introduction

**Figure 1:**
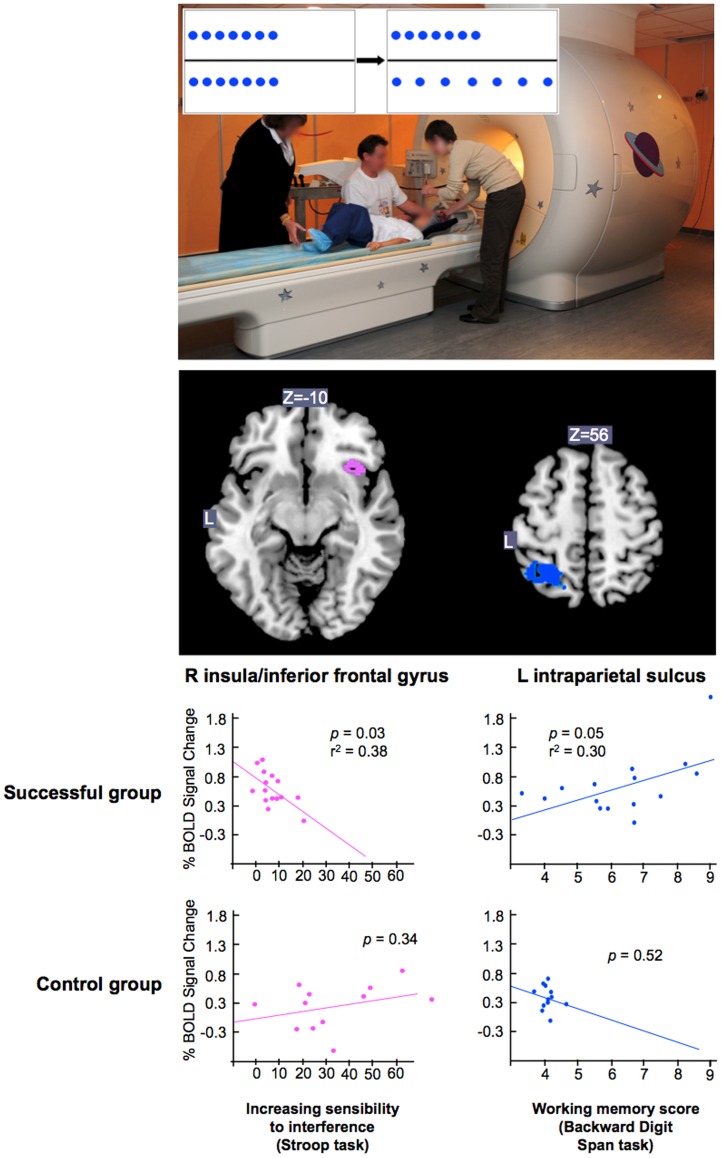
Example of conservation-of-number trial and percentages of explained variance between percentage of fMRI signal change during the conservation task and behavioral scores for the successful and the control groups of children. Lower scores for the Stroop task reflect higher inhibitory control efficiency.

In most situations in the visual environment, when the number of objects increases, a greater portion of space is occupied. Many empirical studies demonstrate that space and numbers are not only strongly associated at a behavioral level but also recruit overlapping cortical regions in the human brain [1], [2], [3], [4]. In situations in which numbers and length conflict (e.g., discrete linear arrays of coins with the same number of coins but different lengths), a fronto-parietal network is recruited [5]. The activation of this network allows one to surpass the spatial bias (i.e., length equals number) to correctly focus on the comparison of numbers [6], [7] (see [8] for a discussion on the involvement of the intraparietal sulcus in numerosity and length processing). This relationship between space and number appears early in cognitive development [9]. Young children tend to believe that the spatial extent of a group of objects reflects the number of objects in that group. Piaget’s famous conservation-of-number task [10] is a classical example of children’s inability to dissociate the number of objects in arrays from their spatial extents. In this task, children first determine whether two rows containing the same number of coins placed in a one-to-one correspondence are equal in number. Once the children have acknowledged this equality, one of the rows is transformed in length but not in number (i.e., the coins in the row are spread apart; see Figure 1), and the children are once again asked whether the two rows contain the same number of coins. Before 7 years of age, children erroneously believe that there are more objects in the longer row. This perceptual error has been reported by numerous developmental studies [11], [12], [13]. Errors made by young children in conservation-of-number tasks have been attributed either to children’s inability to fully grasp the concept of number [10], [14] or to children’s inability to inhibit a misleading perceptual strategy, or the ‘length-equals-number’ heuristic [15]. According to Piaget, children around 7 years of age begin to understand that any visuospatial transformations can be mentally canceled out by using the mirror transformation. Hence, the acquisition of the reversibility of cognitive operations allows children to determine that the two rows of coins contain the same numbers of objects, regardless of any apparent transformations in the conservation-of-number task. Thus, in Piaget’s theory, success in this task proves the solidity of the number concept and represents an important step in children’s acquisition of concrete logicomathematical skills. However, Neo-Piagetian authors have suggested that young children erroneously state that the longer row contains more coins because they fail to inhibit the inappropriate ‘length-equals-number’ heuristic [15], [16], [17]. Indeed, in line with Dempster’s claim that “*conservation and class inclusion have more to do with the ability to resist interference than they do with the child’s ability to grasp their underlying logic”* ([16], p. 15), Neo-Piagetians believe that the critical factor for success in Piaget’s numerical or logical tasks is not only the ability to activate the appropriate logicomathematical strategy but also, and most importantly, the executive ability to inhibit misleading visuospatial heuristics (here, the ‘length-equals-number’ heuristic). According to Neo-Piagetians, cognitive development is supported not only by the ability to acquire knowledge of incremental complexity [14] but also by the ability to inhibit previously acquired knowledge (for recent works regarding the importance of inhibition on cognitive development, see [18], [19], [20]). Note that the two accounts of children’s errors in the conservation-of-number task are not mutually exclusive. Piaget argued that children change their judgment after the coins are spread apart because they use the misleading ‘length-equals-number’ intuition. However, Piaget did not hypothesize, based on this observation, that children specifically need inhibitory control as an executive function of the brain (beyond logicomathematical cognition per se) to succeed at this developmental task.

Recently, an fMRI study investigated the brain areas necessary for succeeding at Piaget’s conservation-of-number task [5]. During a functional imaging session, children were presented with a computerized version of Piaget’s classic conservation-of-number task. The results revealed that the acquisition of number conservation is supported by a parieto-frontal network that is involved in the monitoring of different possible and competitive task responses [21]. Thus, the acquisition of the number conservation principle might be directly related to the efficiency of the inhibitory control that allows one to inhibit erroneous intuitive perceptual heuristic responses.

The present work aims to further investigate the role of executive functions in the numerosity-length conflict. In our previous fMRI study [5], success in the conservation-of-number task elicited activations in the right insula/inferior frontal gyrus, a region critical to inhibitory control [22] and resisting interference [23]. In line with this result, we reasoned that if inhibitory control allows children to surpass the length-equals-number conflict, then the percentage of BOLD signal changes in the insula/inferior frontal gyrus should be related to children’s inhibitory control efficiency, as reflected by children’s performance on an adaptation of the Stroop task (a Stroop-like measure of inhibitory function development [24]). To demonstrate that the level of neural activation in this area is directly related to the efficiency of inhibitory control rather than to other cognitive abilities, we also tested whether the percentage of BOLD signal changes in the right insula/inferior frontal gyrus was related to the children’s ability to manipulate information in working memory in a Backward Digit Span task [25]. Indeed, studies report that both inhibitory control and the manipulation of information in working memory rely on the inferior portions of the frontal lobes [26] (see [27] for a review). A control group consisting of children who were unable to perform the number-length interference items was also included. We reasoned that if inhibitory control allows children to surpass the length-equals-number conflict, then we should find no correlation between children’s performance in the Stroop task and the level of activation in the right insula/inferior frontal gyrus in the control group.

## Materials and Methods

### Participants

Two groups of children were created from the original sample tested in the fMRI study [5]. One group (hereafter referred to as the successful group) consisted of 16 children who accurately performed the number-length interference items (mean age: 9 years ± 21 months; 10 girls). The second group (hereafter referred to as the control group) consisted of 12 children who were unable to accurately perform the number-length interference items (mean age: 6 years ± 8 months; 6 girls). Only right-handed children were included because handedness affects performance on the Stroop task [28]. The local ethics committee (CPP Nord-Ouest III, France) approved the study. These children had no history of a neurological or psychiatric disorder. Written consent was obtained from the parents and the children themselves.

### Behavioral Assessment

The children performed an animal Stroop task [24] and a Backward Digit Span task outside the scanner. In the animal Stroop task, the children performed three conditions (congruent, control, and incongruent), each consisting of 24 farm animal pictures presented on a page. Four farm animals were used in each condition (i.e., cow, duck, pig, and sheep). In all three conditions, participants were instructed to name the body of each animal regardless of the head attached to it. In the congruent condition, the head of each animal corresponded to the correct body. In the control condition, human heads were placed on animals’ bodies. Finally, in the incongruent condition, chimeric animals were shown (e.g., a cow’s head attached to a pig’s body). Reaction times (RTs) were recorded for each condition. The one-to-one correspondence condition in the conservation-of-number task is a baseline congruent condition in which length and number co-vary, whereas the experimental incongruent condition is a condition in which length and number conflict. Therefore, individual interference scores for the Stroop task were computed, following the same cognitive logic, by subtracting the congruent RTs from the incongruent RTs for each child. Lower scores reflect higher inhibitory control efficiency. This interference score reflects the time required to disengage from an automatic answer [29].

Children’s ability to manipulate information in working memory was assessed with the Backward Digit Span subtest of the Wechsler Intelligence Scale for Children [25]. In this task, the children listened to series of discrete digits and were asked to recall the series of digits in the reverse order of presentation. The children first performed two series of two digits. The series of digits were incrementally increased by one digit every two trials. The task was stopped when the children failed to recall two trials with the same number of digits. The working memory score was defined as the number of series correctly recalled.

### Functional Magnetic Resonance Imaging Paradigm

Images were acquired using a 3T MRI scanner (Achieva, Philips Medical System, Netherlands). In the first anatomical session, three-dimensional T1-weighted spoiled gradient images were acquired (field of view (FOV) = 256 mm, slice thickness = 1.33 mm, 128 slices, matrix size = 192×192 voxels). The subsequent fMRI session was conducted with T2*-weighted gradient echo planar images (repetition time of 2 s, echo time of 35 ms, and a flip angle of 80° for 31 axial slices, 3.5 mm thick, with a 224-mm FOV and a 64×64 grid; see Houdé et al., 2011 for a detailed description of the fMRI imaging protocol). Throughout this second MRI session, the children performed Piaget’s conservation-of-number task and were presented with conservation trials. Each trial consisted of two rows, each containing the same number of coins (5, 6, or 7). For each trial, the children were asked to judge the numerical equivalence of two rows when the rows had the same length. After the children responded, the coins in one of the rows were spread apart by apparent movements on the computer screen (see Figure 1). The children were again instructed to judge the numerical equivalence of the two rows when their length, but not their number, differed. The children responded by pressing the “same” button or the “not the same” button on the response box. The question “*Is the number of objects the same in both rows?”* was delivered verbally for each trial. Each trial remained on the screen until the children responded.

### Functional Regions of Interest

Functional regions of interest (fROI) were defined according to a contrast analysis between successful and unsuccessful children. The brain activity of the unsuccessful group of children (i.e., children who made errors on the length-interference items inside the scanner during the conservation-of-number task) was subtracted from the brain activity of the successful group of children (i.e., children who correctly performed the length-interference items inside the scanner during the conservation-of-number task) [5]. This contrast revealed the brain areas that were activated more in the successful group and therefore the brain network necessary to succeed at Piaget’s conservation-of-number task. For each child, we extracted the BOLD values from the significant activations (*p*<.01, False Discovery Rate, with a minimum extent of 50 voxels in clusters). These regions were located in the bilateral ventral and dorsal visual pathways and in the bilateral parieto-frontal network (see Table 1). Posterior activations were identified in the calcarine sulcus and lingual, inferior occipital, and temporal gyri for the ventral pathway and in the cuneus and superior and middle occipital gyri for the dorsal pathway. The bilateral parieto-frontal network included the intraparietal sulcus (IPS), angular gyrus, insula/orbital part of the inferior frontal gyrus, and middle frontal gyrus with the orbital/opercular part of the right middle frontal gyrus. The left precentral and bilateral cerebellum were also more active in successful children.

**Table 1:** MNI coordinates and number of voxels of brain regions more activated in children who succeeded to the length-interference items than in children who were not able to efficiently perform the Piagetian task.

Brain regions of interest	Number of voxels	MNI coordinates		
		X	Y	Z
R Calcarine	6556	30	−56	50
R Temporal poles of the superior and inferior temporal gyri	58	48	22	−36
**L Intraparietal sulcus**	**611**	**−38**	**−56**	**56**
L Precentral gyrus	109	−48	−8	58
L/R Middle frontal gyrus	681	50	22	38
	95	−36	34	40
R Middle frontal gyrus, orbital part	103	42	46	−14
	158	44	58	0
**L/R Insula/inferior frontal gyrus, orbital part**	**186**	**36**	**22**	**−10**
	59	−32	20	−14

Note: L = Left; R = Right.

This network represents the brain regions necessary to surpass the length-numerosity interference.

## Results

### Behavioral Data

We analyzed the RTs from the Stroop task in a one-way (condition: congruent vs. control vs. incongruent) repeated-measures analysis of variance. Note that data from one child of the successful group were excluded due to abnormal longer RTs for the congruent condition (47 s) compared to the control (31 s) and incongruent (36 s) conditions. Consistent with previous findings [24], children required more time to perform the incongruent condition (successful group: 32±9 s; control group : 72±28 s) than the congruent or the control conditions (27±7 s and 25±5 s, respectively, for the successful group, 39±10 s and 50±23 s, respectively, for the control group), *F*(2, 14) = 10.10, *p* = .0005 (successful group) and *F*(2, 11) = 19.9, *p*<.0001 (control group). The mean score of the Backward Digit Span test was 6±2 numbers correctly repeated in a backward order for the successful group, and 4±0.3 numbers correctly repeated in a backward order for the control group. Importantly, we found no significant correlation between the inhibitory control score and the Backward Digit Spans (successful group: *p* = .15; control group: *p* = .73), suggesting that the two tasks reflect different cognitive processes, namely inhibitory control and information manipulation in working memory.

### fROI Data and Behavioral Scores

Regression analyses, which included gender and age as covariates, were computed to determine the degree of relationship between the activity of the fROIs and behavioral performance (i.e., the interference Stroop score and Backward Digit Span, see Table 2).

**Table 2:** *F* Ratio and *p* values of the parameter estimates in the regression analysis between the activity of brain regions necessary to surpass the length-numerosity interference and behavioral tests for children who succeeded to the length-interference items (successful group) and for children who were not able to efficiently perform the Piagetian task (control group).

Brain regions of interest	Behavioral results of the successful group				Behavioral results ofthe control group			
	Stroop		BackwardDigit Span		Stroop		BackwardDigit Span	
	*F*	*p*	*F*	*p*	*F*	*p*	*F*	*p*
R Calcarine	1.60	.23	3.29	.10	4.80	.06	2.29	.17
R Temporal poles of the superior and inferiortemporal gyri	1.29	.28	0.03	.87	0.43	.53	0.04	.85
**L Intraparietal sulcus**	**1.74**	**.21**	**4.73**	.05*	**0.18**	**.68**	**0.46**	**.52**
L Precentral gyrus	2.14	.17	0.69	.42	0.28	.61	0.08	.78
L/R Middle frontal gyrus	1.34	.27	0.53	.48	0.08	.78	0.10	.76
	0.92	.36	0.10	.76	0.04	.84	2.05	.19
R Middle frontal gyrus, orbital part	0.99	.34	0.06	.81	0.03	.86	0.24	.64
	0.26	.62	0.70	.42	1.16	.31	0.07	.79
**L/R Insula/inferior frontal gyrus, orbital part**	**5.95**	.03*	**2.75**	**.13**	**1.01**	**.34**	**1.58**	**.24**
	1.37	.27	2.12	.17	0.07	.79	0.72	.42

Note: L = Left; R = Right.

Note that age and gender were included as covariates in the analyses. *p<0.05.

Regarding the successful group, the main result was a double dissociation pattern of neurocognitive correlations in the two key fROIs from the parieto-frontal network necessary to succeed at the Piagetian conservation-of-number task (see Figure 1). A significant negative correlation was found between the interference scores and the percentage of BOLD signal changes in the right insula/inferior frontal gyrus. Backward Digit Spans positively correlated with the percentage of BOLD signal changes in the left intraparietal sulcus. Critically, Backward Digit Spans did not correlate with the percentage of BOLD signal changes in the right insula/inferior frontal gyrus (*p* = .13), and interference scores did not correlate with the percentage of BOLD signal changes in the left intraparietal sulcus (*p* = .21). It is worth noting that the same pattern of correlations was observed when the interference score was calculated by subtracting control RTs from incongruent RTs [24]. The interference score correlated with the percentage of BOLD signal changes in the right insula/inferior frontal gyrus (*p* = .02), but not with the IPS (*p* = .17).

Regression analyses revealed no correlation between the fROIs and the interference Stroop score or the Backward Digit Span for children in the control group who did not succeed at the conservation task (all *p*>.05, see Table 2).

## Discussion

The current study examined whether success in a well-known Piagetian developmental task (i.e., the conservation-of-number task) is determined, at least partially, by children’s ability to inhibit the length-equals-number heuristic. Piaget’s conservation-of-number task is a challenging task for children younger than 7 years of age. Before this age, children erroneously indicate that longer arrays contain more objects when two arrays with the same number of elements have different lengths. Authors have suggested that executive processes, particularly inhibitory control, are critical for resisting number-length interference [17]. To demonstrate that inhibitory control is needed to accurately perform the conservation-of-number task, we computed the correlations between the percentage of BOLD signal changes in fROIs defined in a previous fMRI study [5] and behavioral performance that reflected either inhibitory control efficiency or the ability to manipulate information in working memory. Children’s inhibitory control efficiency–the extra time needed to process incongruent (e.g., a pig’s head on a duck’s body) or congruent (e.g., a duck’s head on a duck’s body) items in a Stroop task, mirroring the two types of items (length-number conflict vs. length-number covariation) presented in the conservation-of-number task–correlated with the level of neural activation in the right insula/inferior frontal gyrus. Notably, no correlation was observed between the activation in the right insula/inferior frontal gyrus and the ability to manipulate information in working memory.

These results suggest that the right insula/inferior frontal gyrus is specifically involved in the need to resist an interference; thus, this may be a core area of the brain network for the inhibitory control required to accurately perform Piaget’s conservation-of-number task. Given that no correlation was found between the activation of this region and the interference Stroop score of children who did not succeed at the conservation-of-number task (i.e., the control group), we suggest that the level of activation in the right insula/inferior frontal gyrus reflected the inhibitory control needed to correctly perform this Piagetian task. This interpretation is further supported by a meta-analysis showing that the right insula (1) is increasingly recruited with increasing executive efficiency during cognitive development [27], and (2) continues to be strongly involved in the inhibition process during adulthood [30]. Note that, in agreement with the processes that are suspected of being involved in the conservation-of-number task, we only studied the correlations between inhibition and working memory processes and brain activations. One could argue that the activity of the right insula might not be restricted to its role in inhibition. Activity in the right insula is also linked, for example, to the capacity to monitor error [31] and to manage response-challenging situations [32]. Nevertheless, the fact that this region correlated in the present study with inhibition but not with working memory scores strongly suggests that the right insula is involved in resistance to interference situations.

The ability to manipulate numerical information in working memory, as revealed by the Backward Digit Span, was related to the activation of the left IPS in children who succeeded at the Piagetian task. This relation was not present in the control group of children who did not resist the number-length interference. The IPS is classically viewed as a numerical area in adults and children [33], [34], [35], so it is coherent that activation in this area could be related to children’s ability to manipulate numbers. The ability to recall a series of digits in reverse order may be a critical ability that allows children to accurately perform the conservation-of-number task. According to Piaget [10], during the conservation-of-number task, children need to mentally reverse the visuospatial transformation that occurs after the initial equivalence phase to determine that two rows of coins of different lengths possess the same number of elements. Thus, children need to maintain the representation of the two rows of coins of different lengths in working memory while they mentally imagine what the two rows would look like when the coins that are spread apart return to their original position. We note that the IPS is recruited not only in number processing but also in length processing [36] as well as in the transformation of visuospatial mental images (as reviewed by Zacks [37], see also [38]). These results, in conjunction with the findings of the present work, suggest that the activation observed in the IPS when children accurately performed the conservation-of-number task may reflect the ability to imagine the mirror visuospatial transformation of a perceptual transformation of objects (such as the spreading of coins), which is an elementary form of a more formal reversibility (e.g., numerical reversibility).

### Conclusion

In conclusion, the present findings provide the first evidence of a direct relationship between the activation of the right insula/inferior frontal gyrus, inhibitory control efficiency and the ability to resolve a length-numerosity conflict. We further characterized the parieto-frontal network involved in number conservation by providing evidence that the activation of the IPS may be related to the ability to reverse visuospatial transformations and operations. The data reported here, in conjunction with data collected in behavioral [17] and event-related studies [39], add support to the view that inhibitory control is necessary to overcome length-numerosity interference in children. In addition, we provided evidence that Neo-Piagetian and Piagetian accounts of numerical cognitive development are not mutually exclusive. After the visuospatial transformation (the spreading of the coins), children need to inhibit the misleading strategy (i.e., length-equal-number, supported by insula/inferior frontal gyri) and to activate the reversibility of the operation (supported by the IPS) to determine that the two rows possess the same number of elements.
